# RACE DIFFERENCES IN THE RELATIONSHIP BETWEEN HIGH AND VARIABLE BLOOD PRESSURE AND DOMAIN-SPECIFIC COGNITIVE CHANGE

**DOI:** 10.1093/geroni/igad104.2238

**Published:** 2023-12-21

**Authors:** Michael Oliver, Cassandra Morrison, Sondos El-Hulu

**Affiliations:** Belmont University, Nashville, Tennessee, United States; Montreal Neurological Institute, McGill University, Montréal, Quebec, Canada; Belmont University, Nashville, Tennessee, United States

## Abstract

Elevated blood pressure (BP), or hypertension, is a risk factor for several health conditions including Alzheimer’s disease. High BP in early- and mid-life is associated with cognitive decline, whereas research is mixed regarding BP and cognition in late-life. Moreover, hypertension disproportionately affects minority populations. Consequently, the effects of hypertension on cognition may differ by race. The present study investigates the relationship between BP and cognition. 4419 older adults (Black, n=1189; White, n= 3230), with 32116 follow-ups for a maximum of 10-years were included from the Rush Alzheimer’s Disease Center. Results reveal individuals with high BP exhibit significantly greater declines in global cognition compared to normal BP, regardless of race. When stratifying high BP by race, Blacks exhibit greater declines in working memory and perceptual speed, whereas Whites exhibit greater declines in episodic memory, semantic memory, and visuospatial ability compared to normal BP. Blacks with high BP exhibit greater decline in perceptual speed compared to variable BP, whereas no cognitive domain was worse in Whites with high BP compared to variable. When stratifying variable BP by race, Blacks exhibited greater decline in working memory compared to normal BP. Alternatively, Whites exhibited greater decline in episodic memory, semantic memory, working memory, and global cognition compared to normal and high BP counterparts. Our findings reveal racial differences in the relationship between BP and cognitive decline. Specifically, Blacks and Whites with high and variable BP experience cognitive decline in different cognitive domains, which may give insight into differences in disease trajectory and cognitive outcomes.

